# Robotic invasion of operation theatre and associated anaesthetic issues: A review

**DOI:** 10.4103/0019-5049.76577

**Published:** 2011

**Authors:** Prem N Kakar, Jyotirmoy Das, Preeti Mittal Roy, Vijaya Pant

**Affiliations:** Department of Anesthesiology Pain Management and Perioperative Care, Fortis Hospital, Shalimar Bagh, New Delhi, India

**Keywords:** Da Vinci Robotic system, extreme position, surgical robot, telemanipulation

## Abstract

A Robotic device is a powered, computer controlled manipulator with artificial sensing that can be reprogrammed to move and position tools to carry out a wide range of tasks. Robots and Telemanipulators were first developed by the National Aeronautics and Space Administration (NASA) for use in space exploration. Today’s medical robotic systems were the brainchild of the United States Department of Defence’s desire to decrease war casualties with the development of ‘telerobotic surgery’. The ‘master-slave’ telemanipulator concept was developed for medical use in the early 1990s where the surgeon’s (master) manual movements were transmitted to end-effector (slave) instruments at a remote site. Since then, the field of surgical robotics has undergone massive transformation and the future is even brighter. As expected, any new technique brings with it risks and the possibility of technical difficulties. The person who bears the brunt of complications or benefit from a new invention is the ‘Patient’. Anaesthesiologists as always must do their part to be the patient’s ‘best man’ in the perioperative period. We should be prepared for screening and selection of patients in a different perspective keeping in mind the steep learning curves of surgeons, long surgical hours, extreme patient positioning and other previously unknown anaesthetic challenges brought about by the surgical robot. In this article we have tried to track the development of surgical robots and consider the unique anaesthetic issues related to robot assisted surgeries.

## DEVELOPMENT OF SURGICAL ROBOTICS

Of all wounded soldiers in Vietnam War, one third of the total deaths were due to exsanguinating haemorrhage that had the potential to survive if they were treated in time.[1] In 1985, NASA instituted a research program in Telerobotics to develop the technology for the United States Space program.[2] Early developments in Telerobotics were confined to the fields of nuclear, underwater and space applications.[3,4] Relevant studies have also been carried out by the German Aerospace Center[5] and the Japanese Space Agency.[6] Newer fields of application of Telerobotics have emerged during the 80’s and 90’s, such as surgery,[7] rescue,[8] education, people assistance, mining, etc.[9,10] The first documented use of a robot assisted surgical procedure was in 1985 when the PUMA 560 robotic surgical arm was used to take a neurosurgical biopsy.[11] In 1990, the U.S. Food and Drug Administration (FDA) approved the Automated Endoscopic System for Optimal Positioning (AESOP)[12] arm for laparoscopic surgery to achieve precise and consistent movements of the camera during surgery. The first telemanipulative robotic assisted laparoscopic cholecystectomy was performed by Jacques Himpens and Guy Cardiere in 1997 in Brussels, Belgium.[13] Marescaux and Gagner[14,15] performed a robot assisted laparoscopic cholecystectomy between New York City and Strousbourg, France in 2001, with a latency time of 155 msec.

## ADVANTAGES

**Figure 1:**
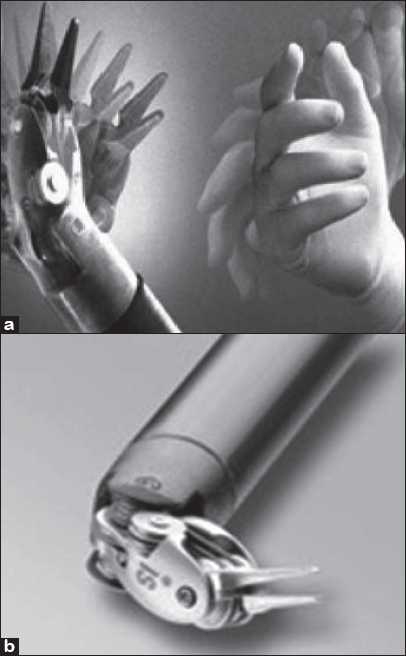
(a) New generation Robotic instruments have 7 degrees of freedom as the human hand (b) EndoWrist’^®^ instrument from Intuitive Surgical [a and b: Courtesy of Intuitive Surgical, Inc, Sunnyvale, CA.]

Robots allow unprecedented control and precision of surgical instruments in minimally invasive procedures and microsurgery [e.g. Trans Oral Robotic Surgery (TORS), natural orifice transluminal endoscopic surgery (NOTES), eye operations, intrauterine fetal surgery]. The robot can filter the surgeon’s hand tremor and scale the movements of the instruments. Present day Robotic surgical systems have 7 degrees of freedom [Figure 1a] just like the human forelimb in contrast to the laparoscopic arm providing only 4 degrees of freedom.[16] Robot motions and tasks are reproducible and are immune to fatigue.[17]

### Limitations of Robot assisted surgery

Concerns about patient safety in the event of Robot malfunction and crash down is an issue that the operating room staff should be aware of. Robots are complex inventions which need a lot of practice and technical expertise. Robotic surgery preparation needs longer operating room time compared to conventional surgeries. Several pieces of equipment, each being extremely bulky require large operating room space.[18] For the anaesthesiologist, invasion of the anaesthesia work space by the robot and difficulty in accessing the patient intra operatively is a concern. The staff must be trained and prepared to quickly detach and remove the robot from the patient in the event of an emergency. Current robotic systems lack tactile feedback from the instruments.[19] Surgeons have to rely on visual clues to modulate the amount of tension and pressure applied to tissues to avoid organ damage. The newly launched da Vinci HD SI system costs $1.75 million. Initial increased operating room setup time and surgical time adds to the cost burden. However, robot assisted surgery has shown to reduce hospital stay by about half and thereby cutting hospital cost by about 33%.[20]

One major obstacle to the telerobotic surgery is the ‘Latent time’, which is the time taken to send an electrical signal from a hand motion to actual visualization of the hand motion on a remote screen. Humans can compensate for delays of less than 200 msec.[21] Longer delays compromise surgical accuracy and safety. Incompatibility with imaging equipments is an area that needs attention.

### Present day surgical robotic systems

Three main types of surgical robots available:

- Supervisory-controlled Robotic Surgery Systems (e. g. the ROBODOC® system from CUREXO Technology Corporation):[22]
  It is the most automated surgical robots available till date. Surgeons can plan their surgery preoperatively in a 3-D virtual space and then execute the surgery exactly as planned in the operating theatre.
- Shared-control Robotic Surgery Systems:
  These robots aid surgeons during surgery, but the human does most of the work.
- Telesurgical devices:
  Here, the surgeon directs the motions of the robot. e.g. the da Vinci Robotic system, the ZEUS Surgical System.

### The da Vinci system

**Figure 2:**
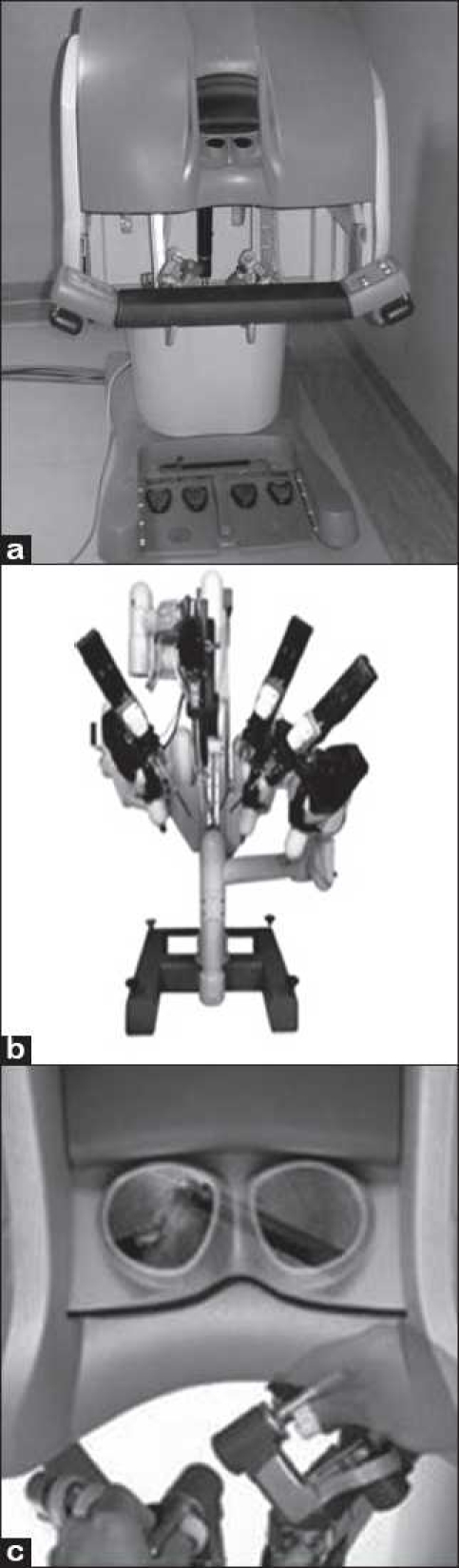
(a) The da Vinci System Surgeon console; (b) the cart with three mounted surgical arms; (c) Joysticks with viewing ports in the console [a-c: Courtesy of Intuitive Surgical, Inc, Sunnyvale, CA.]

A product of Intuitive Surgical, the da Vinci Surgical System [Figure 2a, 2b] falls under the category of telesurgical devices. On July 11, 2000, FDA approved the da Vinci Surgical System for laparoscopic procedures.

Three generations of da Vinci surgical systems have developed so far:[23]

- da Vinci surgical system (1999)
  It consists of three components: The viewing and control console, surgical arm unit (three or four arms depending on the model) and Optical three-dimensional vision tower
- da Vinci S HD surgical system (2006)
  This second generation surgical robot is equipped with wide range of motion of robotic arms and extended length instruments, interactive video displays and touch screen monitor.
- da Vinci Si HD surgical system (2009)
  It has dual console capability to support training and collaboration, advanced 3D HD visualization with up to 10× magnification, ‘EndoWrist’^®^ instrumentation with dexterity and range of motion more than the human hand and ‘Intuitive^®^ motion technology’, which replicates the experience of open surgery by preserving natural eye-hand-instrument alignment.[23]

### Operating with a da Vinci surgical system

**Figure 3:**
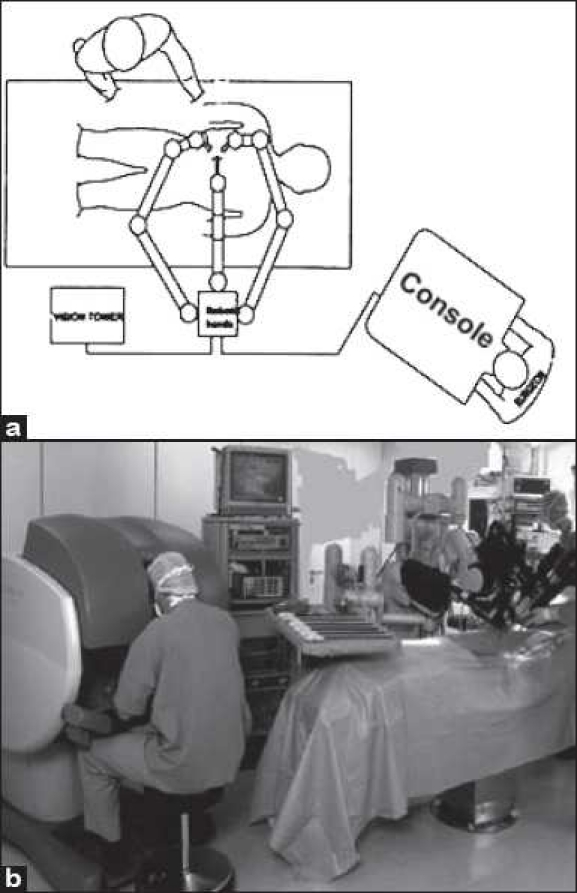
(a) Schematic diagram and (b) Actual photograph showing the arrangement of the operating room in Robot assisted surgery [a: Courtesy of Intuitive Surgical, Inc, Sunnyvale, CA.]

After positioning of the patient, the surgeon makes three or four small incisions (depending on the number of arms the model has) in the patient’s body. One port accommodates two endoscopic cameras in a single rod that provide a stereoscopic image, while the other ports are dedicated for surgical instruments for dissection and suturing. At the console, the surgeon actually looks at two separate monitors; each eye sees through an independent camera channel to create a virtual three-dimensional stereoscopic image. The surgeon uses joystick-like controls located underneath the screen to manipulate the surgical instruments [Figure 2c]. Each time the surgeon moves one of the joysticks, a computer sends an electronic signal to one of the instruments, which moves in sync with the movements of the surgeon’s hands [Figure 3]. To work on a miniature scale, a ‘frequency filter’ eliminates hand tremor greater than 6 Hz and a ‘motion scaling device’ scales down the surgeon’s hand movements[24] up to a ration of 5:1.

### The ZEUS surgical system (Computer Motion Inc)

**Figure 4:**
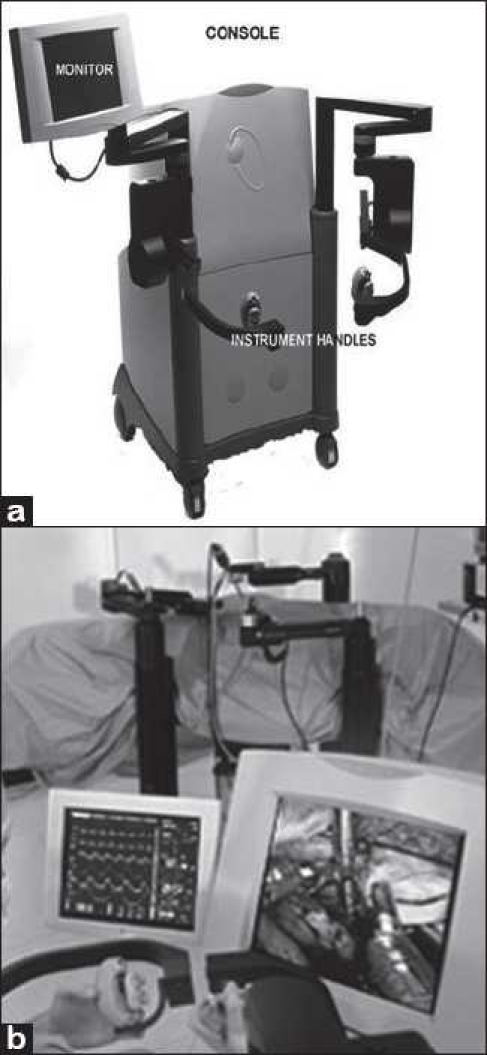
(a)The console of the ZEUS robotic system; (b) the three arms together on patient’s side [a: Courtesy of Intuitive Surgical, Inc, Sunnyvale, CA.]

The ZEUS^®^ surgical system is made up of a surgeon control console [Figure 4a] and three Table-mounted robotic arms [Figure 4b], which perform surgical tasks and provide steady visualization using AESOP technology.[12] In 2003, Intuitive Surgical merged with Computer Motion Inc and the ZEUS system was phased out gradually in favor of the da Vinci system.[25]

## EXAMPLES OF ROBOT ASSISTED SURGERIES

The Table 1 below lists the various commonly performed robot assisted surgeries.

**Table 1:** List of Robot assisted surgeries

Gastrointestinal surgery	Cholecystectomy, splenectomy, Heller myotomy, pancreatectomy, whipple procedure, adrenalectomy, bowel resection, pyloroplasty and antireflux surgery.
Cardiac surgery	Atrial septal defect closures, mitral valve repairs, patent ductus arteriosus ligations, minimally invasive atrial fibrillation surgery, totally endoscopic coronary artery bypass grafting, left ventticular pacemaker lead placement, etc.
Thoracic	Esophageal procedures, resection of esophageal masses, and esophagectomy, including Heller myotomy, resection of mediastinal masses and thymectomy
Neuro surgery	Medical robotics has yet to gain prominence in neurosurgery apart from Cyberknife and ‘NeuroArm’ is the world’s first MRI-Complete surgical robot.

## ANAESTHETIC CONSIDERATIONS

### I) General considerations in all Robot assisted surgeries

#### A) Patient selection

Selection of patients for robot assisted surgery depends on clinical judgement and assessment as to whether the patient could withstand a prolonged period in the extreme position. A history of significant cardiovascular comorbidity, cerebrovascular disease,[26] poor pulmonary function,[27] pulmonary hypertension and glaucoma are considered as independent risk factors for Robot assisted surgeries.[27]

#### B) Intraoperative preparation

Two wide bore intravascular cannulae with extension tubings should be placed to administer anaesthetic drugs and fluids intraoperatively. Antisialogouge agents are used in patients requiring extreme patient positioning. Monitoring includes ECG, noninvasive blood pressure, pulse oximetry, end-tidal CO_2_ and urine output. Urine output is not a good guide of end organ perfusion in procedures involving manipulation and dissection of the urinary tract.[28] A central venous catheter is a reasonable consideration in certain procedures with major fluid shifts as a monitor of central venous pressure (CVP).[29] Similarly, placement of arterial line for continuous arterial pressure measurement is dictated by the nature of surgery and the preoperative functional status of the patient.[28] The patient should be well strapped to the Table to prevent sliding after positioning and a trial run of the final Table position should be done beforehand to check for any strain on monitoring cables, circuit and intravenous tubings. It is important to record the level of CVP and blood pressure after patient positioning and to treat it as baseline as the extreme positioning may render single isolated readings (especially of CVP) inconclusive. The recommended zero reference level for transducer positioning is the angle of Louis. Deep venous thrombosis prophylaxis should be followed strictly as per protocol.

The assisting surgeon creates pneumoperitoneum and makes the ports in the patient’s body. Then the robotic arms are docked into the ports and the chief surgeon starts operating by controlling the robotic arms from the console which is kept a little away from the patient. The size and bulk of the robot over the patient and the significant draping on both the robot and patient make it difficult to access the patient intraoperatively. Some procedures require the patient’s airway to be at a distance from the anaesthesiologist and the anaesthesia machine/monitor. It becomes much more challenging if one-lung ventilation is required, since frequent use of the fiberoptic bronchoscope may be necessary. It is important to have all monitors and safety devices (defibrillator pad, Transesophageal echocardiography (TEE), left precordial stethoscope in pediatric patients to detect inadvertent right bronchial intubation) in place before the Robot is docked. Careful attention should also be given to prevent the robotic arms from injuring the patient.[30] Cameras and light sources should never be kept directly on drapes or patient’s skin.

#### C) Patient positioning

Common patient positions used are steep Trendelenburg with legs apart for prostatectomy, supine or slight lateral decubitus (raising one side 15° to 30°) position for anterior mediastinum pathology, 90° lateral position for hilar mass and lobectomy and a nearly prone position for posterior mediastinal mass. It is difficult to change the patient’s position once the Robot is docked. So, proper patient positioning should be confirmed beforehand with the surgical team. It is highly recommended that the anaesthesiologist is well versed with various patient positions and their implications. Proper padding/cushions over pressure points should be used to avoid tissue and nerve impingement. While using extreme patient positioning, restraints must be used to prevent the risk of anaesthetized patient sliding off the Table.

Extreme patient positioning and pneumoperitoneum can cause endotracheal tube migration into the main stem bronchus. Before docking of the Robot, tube positioning must be confirmed. Insignificant changes in cardiac output or stroke volume were noted[31] in spite of increase in mean arterial pressure and systemic vascular resistance. Cerebral oxygenation was shown to increase slightly provided PaCO_2_ was kept within normal limit.[32,33] IOP can increase on an average 13 mm Hg higher than the baseline. Surgical duration and ETCO_2_ are significant predictors of IOP increase in the Trendelenburg position.[34] Severe oral ulceration and conjunctival burns may occur from reflux of stomach acid onto the face. As a precautionary measure, stomach should be decompressed by oro/nasopharyngeal tube and the patients’ face kept visible intraoperatively.[28]

#### D) Anaesthetic technique

Oxygen, air mixture is used along with inhalational agent and Fentanyl/Remifentanil infusion for maintenance of anaesthesia. In our experience Sevoflurane is the preferred agent in view of its recovery profile and lack of significant central nervous system effects. The anaesthetic issues related to laparoscopic surgeries with creation of pneumoperitoneum are beyond the scope of this article. However we do recommend placement of an epidural catheter and an epidural infusion for not only intra and post operative pain relief but also for the gut volume reduction. Epidural test dose and initial bolus should be given well before patient positioning. Continuous uniform depth of muscle relaxation is of prime importance in avoiding any movements by the patient while the surgical instruments are in place and starting an infusion of muscle relaxant is recommended.

Fluid replacement: Initial fluid loading is inappropriate in extreme patient positioning and in surgeries needing urethral anastomoses.[28] Suction, made up of a mixture of flush (saline), blood and urine, is not a reliable measure of blood loss.[28] In long operations and when there was evidence of excessive blood loss, not tallying with the suction, intraoperative haematocrit may give a rough guide.

Diuresis: Mannitol 1–2 g/kg or Furosemide can be used.[28] The rationale is threefold: to promote urine flow to flush out and maintain urinary tract patency, to conserve renal function, and as a prophylaxis against cerebral swelling in extreme Trendelenburg position.

Cerebral protection: Fluid restriction, maintaining intraoperative ETCO_2_, using minimal insufflation pressures and use of diuretics towards the end of the procedure are some of the techniques commonly employed for avoiding cerebral oedema.

Reversal: Cognitive recovery may be delayed because of the cerebral oedema and raised intracranial tension, especially after a long surgery in steep head down position. So, early discontinuation of anaesthetic agents may be necessary as soon as the Robot is withdrawn. With more experience and skill and reduced operating time, the issue of delayed cognitive recovery may be resolved. There have been reports of stridor after extubation of the trachea, following laryngeal oedema due to prolonged steep Trendelenburg and overjudicious fluid administration.[35] Presence of peri-orbital oedema should alert the Anaesthetist of the possibility of concomitant airway oedema. Maintenance of airway and prevention of aspiration should be taken care of. There are reports of compartment syndrome in the calves after prolonged lithotomy,[36] necessitating routine checks for calf tightness and tenderness.

#### E) Postoperative pain relief

The severity of postoperative pain is less in robot assisted laparoscopic surgeries compared to open procedure. Simple analgesics and opioids as infusions or bolus generally suffice. Epidural analgesia or for that matter anaesthesia is indicated in certain surgeries needing periopertaive sympathetic blockade apart from its use for pain relief.

### II) Important issues related to specific surgeries

#### Cardiac surgery

Robotic surgery may require unprecedented, prolonged one-lung ventilation. This tests the limits of our knowledge and understanding of one lung anaesthesia. Confirmed placement of a left-sided double-lumen endotracheal tube (DLT) is necessary to allow for the single left-sided ventilation required for cardiac exposure. DLT is preferred to Bronchial blockers in robot assisted cardiac surgery because intermittent right lung inflation is necessary for adequate oxygenation during weaning from Cardiopulmonary Bypass (CPB). Moreover, isolation of the right lung may again be necessary to check for bleeding post CPB.[37] Knowledge of TEE is a must in robot assisted surgeries.

#### Thoracic surgery

The principles that apply for thoracoscopic surgery apply for robotic assisted thoracic surgery. A combination of patient position, one lung anaesthesia, and surgical manipulation alter ventilation and perfusion profoundly. Frequently robotic assisted surgeries require insufflation of CO_2_ in the chest (CO_2_ pneumothorax). This may lead to an increase in the airway pressures and haemodynamic instability secondary to decrease venous return and cardiac compliance. The rate of CO_2_ elimination is difficult to match with the rate of CO_2_ absorption and production during one lung anaesthesia as minute ventilation may already be maximized. Iatrogenic injury to the contra lateral pleura can result in occult blood loss and a tension pneumothorax on the dependent chest.

#### Urological procedures

The principles involved are already described in the general section.

#### Paediatric surgery

A left-sided precordial stethoscope placed beforehand monitors for inadvertent right mainstem intubation. Fibreoptic Bronchoscope may be used to verify tube position. In infants, confirming proper endotracheal tube positioning with fluoroscopy may help prevent an airway emergency.[38]

## THE SCENERIO OF ROBOT ASISTED SURGERY IN INDIA

In our country the availability of surgical Robot is limited to only a couple of centers. The costs of the machine as well as the operative cost are the main deterrents to its popularity. Escorts Heart Institute and Research Centre was the first institute in India to acquire a surgical robot (da Vinci surgical system).[39] In India the first robotic urology surgery was performed in April, 2005[40] and first robotic thoracic surgery (thoracoscopic thymectomy) in 2008. Recently, CARE Foundation in collaboration with Indian Institute of Information Technology (IIIT) Hyderabad has undertaken the task of developing indigenous robotic surgical systems. It is envisaged that such systems would be required at large numbers in India in the near future.[41]

## THE FUTURE BELONGS TO ROBOTIC SURGERY: WHY NOT BE A PART OF THE CHANGE!

Robotic surgery is a new and exciting tool that is beginning to see adoption in our mind. We have already entered a new era of practical robotics that will one day rewrite the final frontier of surgical challenges. As anaesthesiologists, we need to be aware of this fast-changing field and how it affects anaesthetic techniques and their delivery. The future of robotic surgery is nearly as promising as the human will to invent better ways of accomplishing delicate medical procedures. The separation of patient from human contact during surgery, may herald the era of ’no infection, no antibiotic’. Recently, the da Vinci Surgical System has been cleared by the FDA for TORS procedures to treat oropharyngeal tumors in adults.[42] Further advances in ‘motion gating technology’ will one day improve surgery on mobile structures, such as the beating heart by creating an image in virtual stillness.[1] It is hypothesized that the surgical robots might one day replace human surgeons in most if not all the surgical procedures. But, are we Anaesthesiologists immune to this possibility? The answer is ‘perhaps not’. A group of researchers at Montreal’s McGill University have invented an anaesthesia robot called “McSleepy” that can act like an anaesthesiologist, analyze biological information and constantly adapts its own behaviour and even recognizes monitoring malfunction.[43] Such is the progress of Medical Robotics.
